# Statistical competencies for medical research learners: What is fundamental?

**DOI:** 10.1017/cts.2016.31

**Published:** 2017-05-09

**Authors:** Felicity T. Enders, Christopher J. Lindsell, Leah J. Welty, Emma K. T. Benn, Susan M. Perkins, Matthew S. Mayo, Mohammad H. Rahbar, Kelley M. Kidwell, Sally W. Thurston, Heidi Spratt, Steven C. Grambow, Joseph Larson, Rickey E. Carter, Brad H. Pollock, Robert A. Oster

**Affiliations:** 1 Division of Biomedical Statistics & Informatics, Department of Health Sciences Research, Mayo Clinic, Rochester, MN, USA; 2 Department of Emergency Medicine and Center for Clinical and Translational Science and Training, University of Cincinnati, Cincinnati, OH, USA; 3 Department of Preventive Medicine, Northwestern University, Evanston, IL, USA; 4 Department of Population Health Science and Policy, Center for Biostatistics, Icahn School of Medicine at Mount Sinai, New York, NY, USA; 5 Department of Biostatistics, School of Medicine, Indiana University, Indianapolis, IN, USA; 6 Department of Biostatistics, School of Medicine, University of Kansas Medical Center, Kansas City, KS, USA; 7 Department of Internal Medicine, McGovern Medical School, Division of Clinical and Translational Sciences, University of Texas Health Science Center at Houston, Houston, TX, USA; 8 Department of Biostatistics, University of Michigan, Ann Arbor, MI, USA; 9 Department of Biostatistics and Computational Biology, University of Rochester, Rochester, NY, USA; 10 Department of Preventive Medicine and Community Health, The University of Texas Medical Branch, Galveston, TX, USA; 11 Department of Biostatistics and Bioinformatics, Duke University School of Medicine, Durham, NC, USA; 12 Department of Public Health Sciences, University of California, Davis, Davis, CA, USA; 13 Department of Medicine, Division of Preventive Medicine, University of Alabama at Birmingham, Birmingham, AL, USA

**Keywords:** Statistical competency, team science, Clinical and Translational Science, Public Health, Evidence-Based Medicine

## Abstract

**Introduction:**

It is increasingly essential for medical researchers to be literate in statistics, but the requisite degree of literacy is not the same for every statistical competency in translational research. Statistical competency can range from ‘fundamental’ (necessary for all) to ‘specialized’ (necessary for only some). In this study, we determine the degree to which each competency is fundamental or specialized.

**Methods:**

We surveyed members of 4 professional organizations, targeting doctorally trained biostatisticians and epidemiologists who taught statistics to medical research learners in the past 5 years. Respondents rated 24 educational competencies on a 5-point Likert scale anchored by ‘fundamental’ and ‘specialized.’

**Results:**

There were 112 responses. Nineteen of 24 competencies were fundamental. The competencies considered most fundamental were assessing sources of bias and variation (95%), recognizing one’s own limits with regard to statistics (93%), identifying the strengths, and limitations of study designs (93%). The least endorsed items were meta-analysis (34%) and stopping rules (18%).

**Conclusion:**

We have identified the statistical competencies needed by all medical researchers. These competencies should be considered when designing statistical curricula for medical researchers and should inform which topics are taught in graduate programs and evidence-based medicine courses where learners need to read and understand the medical research literature.

## Introduction

As information and data in health research become ever more complex, the ability to draw inferences from those data becomes more challenging. Drawing inferences from information is the core goal of statistics, and the specialty of biostatistics has evolved to focus on the application of statistical methods to solving biological problems. The field of biostatistics continues to develop, taking advantage of new statistical methods and rapidly increasing computational power. As biostatistics in medical research has become ubiquitous, it is increasingly essential for medical professionals and medical researchers to be literate in statistics, but the requisite degree of literacy may not be the same for every statistical competency or for every learner [1].

Several specialties have evolved statistical competencies for their learners, including Clinical and Translational Science (CTS), Public Health (PH), Evidence-Based Medicine (EBM), and Graduate Medical Education (GME). Competency can be defined as the ability or skill to do something successfully or efficiently [2]. In the context of education, competencies are used to define a field and in designing curricula for learners in that field. Core competencies specify essential topics that all students need to learn as fundamental, in contrast to topics that are more specialized in nature. The Education Key Function Committee of the Clinical and Translational Science Award’s (CTSA) National Consortium developed a set of competencies for Master’s degree-level training [3], inclusive of statistical competencies. PH has a great deal of overlap with CTS with regard to statistical competencies and the statistical competencies for PH [4] and CTS [3] have been combined [5] and continue to evolve [1]. In contrast to CTS and PH that seek to train independent medical researchers, EBM and GME are concerned with training medical professionals to critically evaluate the medical research literature and to incorporate research findings into practice-based learning environments [6]. Published statistical competencies for EBM and GME do not include as much detail as those for CTS and PH, and are generally much more limited in scope [4, 7, 8].

Although statistical competencies have been defined, a major finding by Oster *et al*. [1] was the need to categorize the competencies into those that are either fundamental or specialized for different learner groups. Although not an *a priori* question in that study, respondents indicated that only some of the competencies were needed for all trainees. Further, during a convened discussion of statistical competencies, members of the Association for Clinical and Translational Statisticians (ACTStat) concluded that the terminology used to describe the competencies was the basis of their consideration as fundamental to a learner’s needs.

Reflecting on Bloom’s taxonomy [9–11] and the emerging era of team science, ACTStat discussants proposed that the competencies should be phrased to reflect the fundamental needs of medical research learners, cognizant that some learners would want or need to excel in specific competency areas. The verbs ‘propose’ and ‘evaluate’ were suggested to indicate a high level of independence in applying a competency. A working group subsequently convened to rewrite the competencies in order to reflect the role of the health research learner within the research team. Changes to wording were made to frame competencies at the most foundational knowledge level, recognizing that some learners would require a higher level of mastery for more specialized topics. The group deliberately selected the verb ‘understand’ to describe the foundational knowledge level, even though this term is often excluded from use in describing competencies because it is difficult to evaluate understanding [12]. The competencies refined by this working group are shown in online Supplementary Material S2 together with their original phrasing and sources.

The aim of the present study was to evaluate the revised statistical competencies for medical research learners to better understand the degree to which each competency is fundamental for all learners, as opposed to the degree to which the competency is considered appropriate for more specialized training.

## Methods

The present study was approved by the Mayo Clinic Institutional Review Board. An electronic survey was emailed in 2015 to all members of 4 professional organizations: (1) the American Statistical Association’s Section on the Teaching of Statistics in the Health Sciences; (2) the ACTStat; (3) the Association of Clinical and Translational Science’s Biostatistics, Epidemiology, and Research Design (BERD) Special Interest Group; and (4) the former CTSA’s BERD Key Function Committee. As the third group replaced the fourth on the dissolution of the CTSA National Consortium’s Key Function Committees, the 2 BERD groups were combined for this analysis.

The survey was implemented in the Research Electronic Data Capture (REDCap) system [13]. Email messages were sent to all members of the 4 organizations on days 0, 7, and 14. The cover letter, which was repeated with each successive mailing, included bullet points describing who should respond, as well as bullet points highlighting the ways in which the study’s results might help faculty and institutions. The cover letter was tailored to each group, including the names and positions of leaders of that group who had previewed the survey and agreed to the mailing. As responses were anonymous, we could not exclude potential multiple responses from the same individual. The likelihood of this was low given that the requests were distributed simultaneously to all groups, and the cover letter requested that individuals complete the survey only 1 time even if solicited as a member of multiple groups. We included responses from Ph.D. and Sc.D. educators who had taught statistics to health researchers in the previous 5 years. We excluded responses from those who were not trained at the doctoral level, who had taught only undergraduates not in pre-med programs during the previous 5 years, or who had taught only statistics or biostatistics students during the previous 5 years.

Respondents were presented with a list of 24 competencies, and were asked to rate each competency on a semiquantitative 5-point Likert scale anchored at 1 for ‘fundamental’ and at 5 for ‘specialized.’ Fundamental was defined for respondents as ‘every CTS learner needs to achieve this competency,’ and specialized was defined as ‘only advanced learners in some areas need to achieve this competency.’ Respondents could also choose to exclude the competency as being entirely unnecessary for CTS learners. As the purpose of this study was to determine what is fundamental, such exclusions were combined with ‘specialized’ for numeric scoring.

### Statistical Methods

Characteristics of respondents are summarized using frequencies and proportions or medians and interquartile ranges as appropriate. We estimated the number and percentage of respondents rating each competency as fundamental (defined as a response of either 1 or 2). We also created frequency histograms of the percentage responding in each category (1=fundamental to 5=specialized; shown for all competencies in the online Supplementary Material S1) with the exact 95% confidence interval. We did not declare an *a priori* cutoff for a competency to be considered fundamental. Instead, we used the lower limit of the 2-sided 95% confidence interval for the proportion of respondents rating a competency as either 1 or 2 as a guide to determine fundamental competencies. If the lower limit was greater than 50%, the competency was considered fundamental. Consequently, our results represent inferences for the population from which this sample was drawn.

## Results

The survey was sent to between 605 and 971 unique people (the exact number remains unknown because some respondents were members of multiple groups and may have had multiple email addresses). The number of individuals meeting eligibility criteria was also less than this, but unknown for similar reasons. There were 112 eligible responses from Ph.D. and Sc.D. educators who indicated that they had taught statistical topics to health research learners in the previous 5 years. Of these, 28 respondents reported belonging to both the ACTStat and BERD groups, representing 88% of eligible ACTStat responses and 62% of eligible BERD responses.

Demographic and teaching characteristics of the respondents are shown in Table 1. Forty-two percent were female, and the median time since highest degree achieved was 18 years. Respondents tended to be of more senior academic rank, with 45% holding an appointment as Professor and only 23% as Instructor, Assistant Professor, or other. Fifty-one percent of respondents were trained as biostatisticians, 29% were trained as statisticians, 10% were trained as epidemiologists, and 11% were trained in other related areas. During the previous 5 years, 83% of respondents taught physicians engaged in research and 69% taught medical students or physicians not engaged in research.

**Table 1:** Demographics and characteristics of the respondents

Variables	Number (%) of 112
Female	45 (40.2%)
Years since highest degree*	18 (12, 31)
Academic rank	
Professor	50 (44.6%)
Associate Professor	36 (32.1%)
Assistant Professor	19 (17.0%)
Instructor/other	7 (6.3%)
Respondent discipline	
Biostatistician	57 (50.9%)
Statistician	32 (28.6%)
Epidemiologist	11 (9.8%)
Other related areas	12 (10.7%)
Learners taught	
Undergraduate students	45 (40.2%)
Master’s candidates	103 (92.0%)
Doctoral candidates	96 (85.7%)
Medical students	52 (46.4%)
Physicians in research	93 (83.0%)
Other medical professionals	78 (69.6%)
Early career nonstatistical faculty	74 (66.1%)
Disciplines taught	
Pre-Med	23 (20.5%)
Medicine	77 (68.8%)
Epidemiology	77 (68.8%)
Biostatistics or statistics	90 (80.4%)
Other public health	63 (56.3%)
Clinical and Translational Science	73 (65.2%)
Other health related	67 (59.8%)

* Median (25th percentile, 75th percentile).

In all, 19 of the 24 competencies were considered fundamental by having a confidence interval entirely above 50%. The competencies are ranked in Table 2 by the degree to which they were considered fundamental by respondents. The exact wording used for each competency is also shown.

**Table 2:** Number (%) of respondents rating each competency as 1 or 2 (1 was fundamental, 3 was neutral, and 5 was specialized)

Rank	Competency	Percent fundamental	95% exact confidence interval
1*	Assess sources of bias and variation in published studies and threats to study validity (bias) including problems with sampling, recruitment, randomization, and comparability of study groups	94.6	88.7, 98.0%
2	Recognize limitation in statistical competency and realize when it would be best to involve a professional statistician	92.9	86.4, 96.9%
2*	Identify the strengths and limitations of study designs for addressing a clinical or translational research question	92.9	86.4, 96.9%
4	Communicate research findings for scientific and lay audiences	89.3	82.0, 94.3%
5*	Understand the basic principles and practical importance of probability, random variation, commonly used statistical probability distributions, hypothesis testing, type I and type II errors, and confidence limits	87.4	79.7, 92.9%
6	Understand the value of data quality and data management	87.3	79.6, 92.9%
7	Understand the reasons for performing research that is reproducible from data collection through publication of results	85.6	77.6, 91.5%
8	Understand appropriate methods for data presentation, especially effective statistical graphs and tables	82.9	74.6, 89.4%
8	Distinguish between variable types (eg, continuous, binary, categorical) and understand the implications for selection of appropriate statistical methods	82.9	74.6, 89.4%
8*	Understand the potential misinterpretation of results in the presence of multiple comparisons	82.9	74.6, 89.4%
11	Evaluate size of the effect with a measure of precision	82.1	73.8, 88.7%
12*	Understand issues relating to generalizability of a study, including sampling methods and the amount and type of missing data	80.9	72.3, 87.8%
13	Evaluate the impact of statistics on ethical research (eg, an inadequate power calculation may mean it is unethical to ask subjects to consent to a study) and of ethics on statistical practice	79.5	70.8, 86.5%
14	Compute descriptive and simple inferential statistics appropriate for the data and research question	76.8	67.9, 84.2%
15	Understand the components of sample size, power, and precision	71.4	62.1, 79.6%
16*	Understand the need to address loss to follow-up	68.8	59.2, 77.3%
17*	Understand the concepts and bias implications of reliability and validity of study measurements and evaluate the reliability and validity of measures	66.7	57.1, 75.3%
18	Evaluate potential violations of the assumptions behind common statistical methods	65.2	55.6, 73.9%
19	Identify when clustered, matched, paired, or longitudinal statistical methods must be used	64.9	55.2, 73.7%
20† ^,^ *	Understand the concepts of sensitivity, specificity, positive and negative predictive values, and receiver operating characteristic curves	59.1	49.3, 68.4%
21†	Understand the purpose of data and safety monitoring plans	49.5	39.9, 59.2%
22†	Identify appropriate methods to address potential confounding and effect modification	47.7	38.1, 57.5%
23† ^,^ *	Understand the purpose of meta-analysis and its place in the hierarchy of evidence	34.2	25.5, 43.8%
24†	Understand the uses, importance, and limitations of early stopping rules in clinical trials	18.0	11.4, 26.4%

* Competencies required in order to evaluate ‘design-specific susceptibility to error,’ needed for literacy regarding evidence within the Scientifically Informed Medical Practice and Learning Model.

† These competencies can be considered important for training but are not fundamental for all learners according to this survey.

The competency considered most fundamental was assessing sources of bias and variation in papers, which was rated 1 or 2 by 95% of respondents. Recognizing one’s own limitation with regard to statistics and identifying the strengths and limitations of study designs were rated 1 or 2 by 93% of respondents. These, combined with communicating research findings (89% fundamental), understanding the basic principles of biostatistics (87%), understanding the value of data quality and data management (87%), and understanding why research needs to be reproducible (86%), represented the highest rated competencies. Ratings did not differ between respondent groups (results not shown).

## Discussion

With this survey, we have identified a set of 19 statistical competencies that can be considered fundamental for all medical research learners. Three different groups of educators were included in this survey, each offering a similar perspective. This suggests that these results are stable and may be viewed as comprehensive for medical research learners. When reviewing the competencies, it is critical to remember that they are written for all medical research learners. For those learners who anticipate taking on specific roles, such as leading research in particular areas, there may exist the need for a greater level of mastery of 1 or more topics than might otherwise be required.

### What is Fundamental?

The degree to which some competencies were viewed as fundamental differed substantially from the results of the study by Oster *et al*. [1]. We attribute the majority of these differences to intentional changes in the wording of the competencies, although sampling differences may also contribute. In the online Supplementary Material S2, we show changes in wording for all the competencies.

**Fig. 1:**
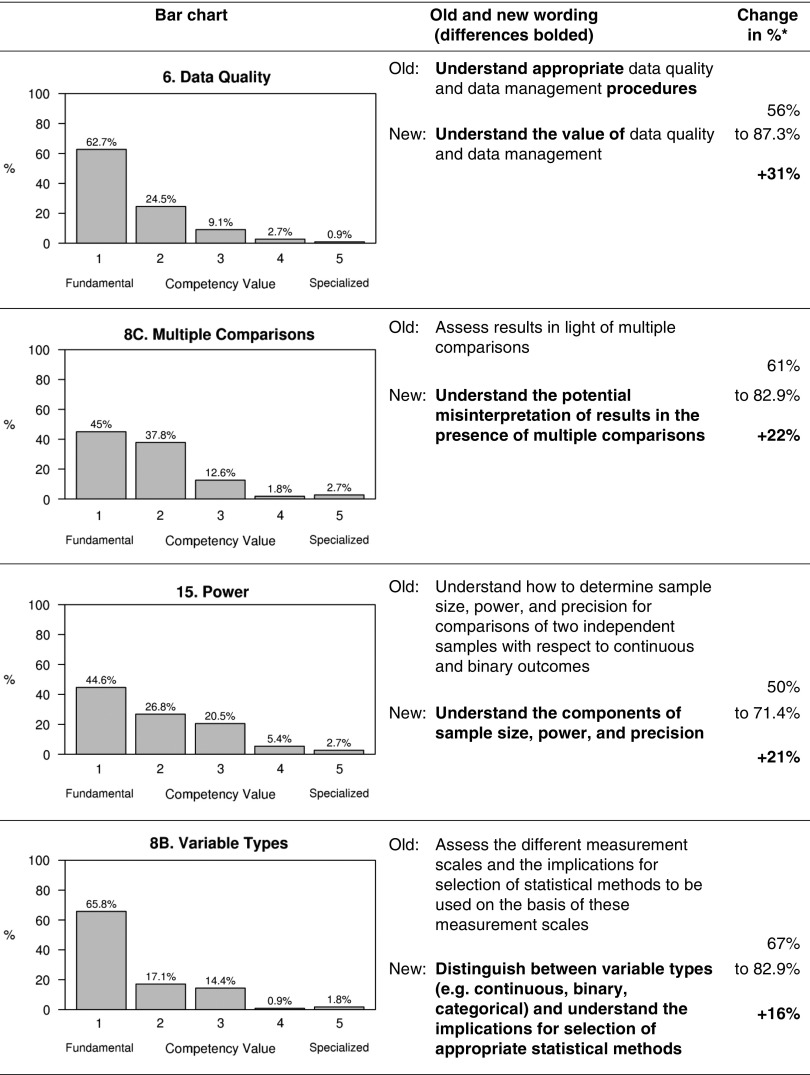
Bar chart, difference in competency wording, and change in percentage from Oster *et al*. [1] for the 4 competencies with the highest positive change. *Differences rounded to the nearest whole percentage.

In most cases, the intent of the change in wording was to shift the emphasis from the learner’s ability to perform a task independently to the learner’s ability to critically evaluate the medical research literature or communicate effectively with a statistician. Fig. 1 shows the 4 competencies that changed most toward being fundamental from Oster *et al*. [1] to this survey, together with the change in the wording. The wording change was intended to shift the emphasis toward the learner’s needs within a team science environment that is inclusive of a statistician. The largest shift was seen for understanding the value of data quality and data management. Interestingly, understanding the potential misinterpretation of results in the presence of multiple comparisons was less affected by the change in wording, highlighting a critical need for medical research learners to be prepared to interpret statistical results appropriately both in their own work and when reading the medical research literature.

**Fig. 2:**
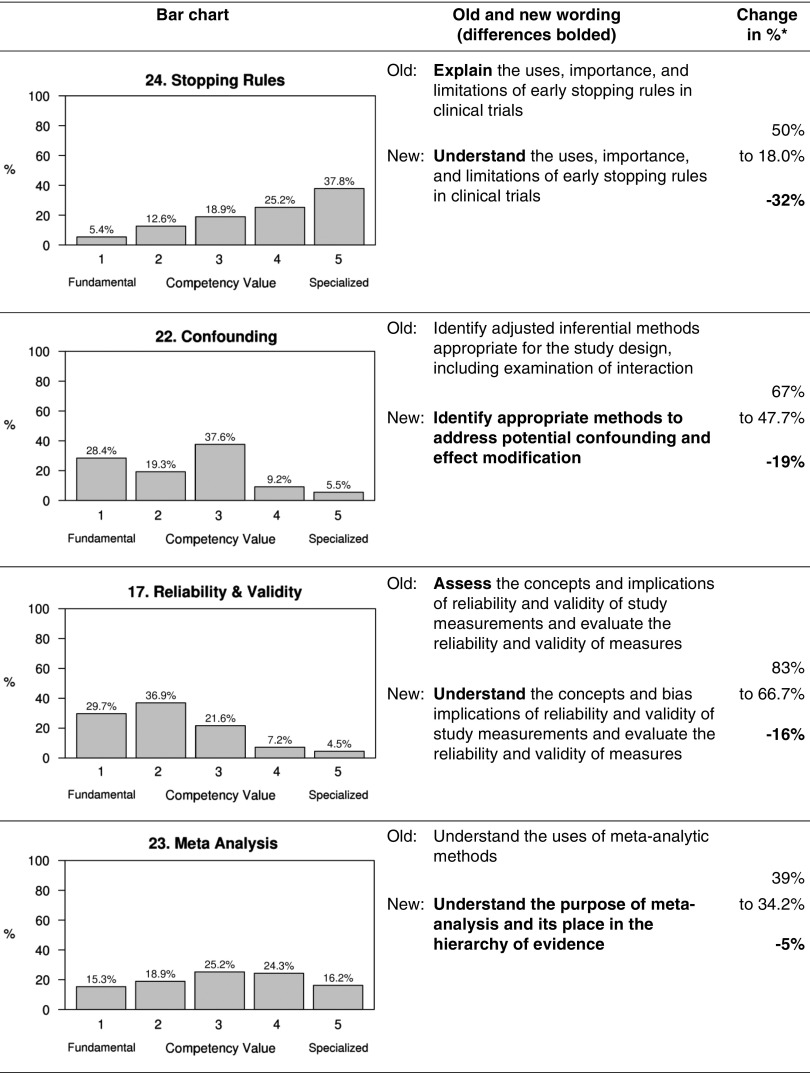
Bar chart, difference (bolded) in competency wording, and change in percentage from Oster *et al*. [1] for the 4 competencies with the highest negative change. *Differences rounded to the nearest whole percentage.

Our results show a decrease in the importance of several topics when compared with Oster *et al*. [1]. The 4 with the greatest decrease in importance are shown in Fig. 2. For topics related to reliability and validity, the wording change was intended to emphasize the skills needed for medical research learners to function within team environments. That competency remains fundamental despite the decrease in percentage. For the other 3 competencies showing a decrease in importance as being fundamental, the wording was intended to reframe the competency because of ambiguity seen with the previous wording. For ‘meta-analysis,’ the change in wording was intended to shift the focus from writing a paper to critically evaluating the medical research literature. In the study by Oster *et al*. [1], learners who wished to become a primary investigator (PI) were reported to need this competency by 39% of respondents compared with 6% for learners who wished to become an informed reader of the literature. Unless a learner wishes to undertake a study involving meta-analytic methods, it might be argued that curricula around meta-analysis should be limited to a general understanding of the strengths and weaknesses of different approaches to research so as to facilitate interpretation of the literature. Similarly, for stopping rules, the change was implemented to de-emphasize the value of a learner carrying out this work independently. For both competencies, the term ‘understand’ may have been perceived to be at a higher level than intended. We suggest that health researchers be sufficiently familiar with these concepts to react appropriately to the literature or to incorporate needed expertise into a research team. Indeed, a better term for this level of competency might be ‘describe.’

A team science approach for biostatistics is described in the study by Perkins *et al*. [14]. Many of the competencies we describe focus on the learner’s understanding and appreciation for activities carried out by the statistician on the team. There are, however, some competencies that all learners should be able to perform independently. These include activities needed for interpreting the research literature, such as bias ascertainment or assessment of study design. In addition, learners should have the skills to appropriately compute simple statistics such as those used to describe a study cohort or to compare 2 study groups, and to recognize when study design will impact analysis such as when data are paired or clustered. Within the context of team science, the investigator who is able to conduct simple statistical analyses and understand the impact of study design is better able to understand the underlying data and its structure than an investigator who cannot. Mastery of the fundamental competencies ensures that medical research learners who engage in team science with a quantitative partner will have the appropriate skill set to collaborate and succeed in research.

### Broader Implications

Learners who understand the assumptions and pitfalls of the statistical methods used to generate scientific evidence in their field will be more informed consumers of the literature [15]. The inclusion of statistical competencies focused on ensuring the learner can appropriately read and evaluate the health research literature highlights the overlap between the CTS, PH, EBM, and GME competencies. Indeed, our study demonstrates a limitation in current descriptions of competencies needed to evaluate the literature in EBM and GME. Within EBM, there is a cognitive skill associated with applying knowledge of study designs and statistical methods to the evaluation of the research literature [6], but these are not described in the detail we offer. Within GME, these elements are missing. Silva and Wyer [16] describe a need for literacy regarding evidence within the Scientifically Informed Medical Practice and Learning Model, highlighting the need to evaluate ‘design-specific susceptibility to error.’ Our proposed competencies prioritize the evaluation of study design and statistical methods with respect to potential bias (starred competencies in Table 2). This list provides details regarding bias assessment that can be used to augment the EBM and GME competencies related to a learners’ ability to review and evaluate the research literature [17].

We lack information on what competencies are currently taught with any consistency in CTS degree programs [1]. With the current emphasis on flexible training programs that meet the needs of the learner, the set of competencies we propose could be used to personalize coursework on the basis of incoming competency. Such a process could be facilitated by a validated assessment instrument to objectively score mastery of these competencies. Degree programs and instructors could also utilize our findings to assess curricular offerings so that all competencies are included and, where needed, courses could be modified to target an appropriate competency level. Finally, we recommend that the Clinical and Translational Science Awards Consortium, training grants through the National Institutes of Health, the Council on Education for Public Health, and the Accreditation Council for Graduate Medical Education, among others, use our results to update the statistical competencies for medical research learners and medical professionals.

### Context for Wording

In developing the revised competency wording assessed in this study, our workgroup considered the verb ‘understand’ in the context of the general level of verbs as described in Bloom’s taxonomy [9–11]. There is much disfavor associated with the verb ‘understand’ [12]. Although acknowledged by our team when wording the competencies, the use of this term is justifiable in part due to the different roles the learner may take. Consider the 2001 revised version of Bloom’s taxonomy [10], which included a knowledge dimension (factual, conceptual, procedural, and meta-cognitive knowledge) to which the cognitive process dimension might be applied. The cognitive process was a revision of the original taxonomy (remember, understand, apply, analyze, evaluate, create) [10]. The idea that the meaning of a competency can change with the learner’s role is not new, and relates to the knowledge dimension. For instance, when a learner is reading the literature or working within a team that includes a statistician, the learner may need only factual or conceptual knowledge to ‘understand,’ whereas procedural knowledge would be needed to carry out a study independently.

The verb ‘understanding’ represents the second level of the revised cognitive process and spans the knowledge dimension. Those competencies that include this verb also span the knowledge dimension depending upon the role of the learner. In addition, the competency ‘evaluate the size of the effect with a measure of precision’ is also within the ‘understand’ cognitive process, but the wording places it explicitly at the level of conceptual knowledge as learners are not always expected to do this when engaging in a research project. Of note, with the current language, none of the statistical competencies can be included in ‘remember,’ the lowest level cognitive process.

The 2-way consideration of cognitive process and knowledge can be helpful in considering competencies that represent higher cognitive processes. The communication competency (communicate research findings for scientific lay audiences) represents the highest cognitive process, reflecting that the learner must combine study design and statistical knowledge with the methods and results of the study in order to procedurally communicate findings; ‘combine’ is the highest level cognitive process. This was closely related to identifying the strengths and limitations of study designs for addressing a clinical or translational research question. This competency also represents procedural knowledge, but is associated with the ‘evaluate’ cognitive process; for this topic, the procedural knowledge level is likely required even when the learner is reading the literature. Similarly, the ‘assess sources of bias and variation’ competency is in the ‘evaluate’ cognitive process, as are ‘evaluate the impact of statistics on ethical research’ and ‘evaluate potential violations of the assumptions.’ All of these competencies may have a different level on the knowledge dimension based upon the learner’s role in the team. For example, competencies related to identifying the appropriate statistical method are important when the learner is reading the literature, but learners are not expected to take the lead on these topics when functioning within a research team.

### Strengths and Limitations

This study is the first broad survey of educators who are experienced in the practice of biostatistics and who teach medical researchers the statistical skills required to succeed in their research careers. The survey included 4 national, professional organizations for statisticians. We acknowledge that our response rate appears to be low, although the overall response rate is unknown because the precise number of targeted individuals who were eligible for the survey is not known. A lower response rate may be partly attributed to our decision to outline inclusion criteria in the email sent to potential participants and to the self-exclusion of ineligible respondents. Those not active in teaching, those without a doctoral degree, and those who did not teach graduate health science learners were unlikely to open the survey. Although the respondents may not include all eligible individuals, there is no reason to believe that that our sample represents a particularly biased cohort. As the survey was anonymous, we were unable to exclude potential multiple responses from the same individual, although measures were taken to decrease this possibility. This study focused only on responses from those who teach statistics, and the competencies focused only on what should be taught. Follow-up studies might inquire as to competencies that ought not to be taught to health learners who are not pursuing a research career, competencies specific to the role of principal investigator, and competencies for clinical research professionals. Future studies should include obtaining the perspectives of investigators and other medical professionals who successfully engage in clinical or translational science. We also note that, although we utilized established statistical competencies in the survey, the application of statistical principles to health research is rapidly evolving in the era of ‘data science.’ Future studies will be needed to re-assess competencies in the light of evolving research practices, such as assessing whether course offerings have evolved to meet learners’ needs.

## Conclusions

An important function of those teaching nonstatisticians about statistics should be to ensure that competencies mirror those required for a learner to effectively engage in team science by the time they complete their training. We have built upon a previously published list of statistical competencies to demonstrate applicability to medical research learners. The changes in wording identified the most fundamental level of competency needed to read the medical research literature and to engage in team science. Our findings are robust to different groups of statistical educators from many outstanding academic institutions. Our results also provide insights into additional statistical competencies that might be of benefit to different medical professionals. From the point of view of statisticians who responded to this survey, we conclude that this list of competencies for statistics for medical research learners can now be considered comprehensive.

## Supplementary material

For supplementary material accompanying this paper visit https://doi.org/10.1017/cts.2016.31. click here to view supplementary material
