# Supplementary material for: Statistical competencies for medical research learners: What is fundamental?
Source: J Clin Transl Sci. 2017 May 9;1(3):146–52. doi: 10.1017/cts.2016.31 (PMC5647667; doi:10.1017/cts.2016.31)
Supplement: Supplementary file 1 [file S2059866116000315sup.zip › S2059866116000315sup002.docx]

Appendix 2: Original source and wording modifications for all competencies, ordered as in Table 2.

| **Rank** | **Original source(s)*** | **Oster et al (2015) wording, unless otherwise specified** | **Current wording** |
| --- | --- | --- | --- |
| 1 | CTSA, Publication guidelines | Assess sources of bias and variation in published studies and threats to study validity (bias) including problems with sampling, recruitment, randomization, and comparability of study groups | Assess sources of bias and variation in published studies and threats to study validity (bias) including problems with sampling, recruitment, randomization, and comparability of study groups [no change] |
| 2a | CTSA | **Collaborate with biostatisticians in the design, conduct, and analyses of clinical and translational research** | **Recognize limitation in statistical competency and realize when it would be best to involve a professional statistician** |
| 2b | CTSA, Publication guidelines | **Assess** study designs for addressing a clinical or translational research question | **Identify the strengths and limitations** of study designs for addressing a clinical or translational research question |
| 4 | CTSA, Public Health, Publication guidelines | Communicate research findings for scientific and lay audiences | Communicate research findings for scientific and lay audiences [no change] |
| 5 | CTSA, Public Health | **Assess** the basic principles and practical importance of probability, random variation, systematic error, sampling error, measurement error, commonly used statistical probability distributions, hypothesis testing, type I and type II errors, and confidence limits | **Understand** the basic principles and practical importance of probability, random variation, commonly used statistical probability distributions, hypothesis testing, type I and type II errors, and confidence limits [shortened] |
| 6 | First survey | **Understand appropriate** data quality and data management **procedures** | **Understand the value of** data quality and data management |
| 7 | First survey | Understand the reasons for performing research that is reproducible from data collection through publication of results | Understand the reasons for performing research that is reproducible from data collection through publication of results [no change] |
| 8a | First survey | Understand appropriate methods for data presentation, especially effective statistical graphs and tables | Understand appropriate methods for data presentation, especially effective statistical graphs and tables [no change] |
| 8b | Public Health | Assess the different measurement scales and the implications for selection of statistical methods to be used on the basis of these measurement scales | **Distinguish between variable types (e.g. continuous, binary, categorical) and understand the implications for selection of appropriate statistical methods** |
| 8c | Publication guidelines | Assess results in light of multiple comparisons | **Understand the potential misinterpretation of results in the presence of multiple comparisons** |
| 11 | Publication guidelines | **Assess** size of the effect with a measure of precision | **Evaluate** size of the effect with a measure of precision |
| 12 | Publication guidelines | Assess the study sample, including sampling methods, the amount and type of missing data, and the implications for generalizability | **Understand** **issues relating to generalizability** **of a study, including sampling methods and the amount and type of missing data** |
| 13 | BERD SIG | [Developed for this survey] | Evaluate the impact of statistics on ethical research (e.g. an inadequate power calculation may mean it is unethical to ask subjects to consent to a study) and of ethics on statistical practice (e.g. collecting valid data from consenting subjects while maintaining privacy). |
| 14 | CTSA, Public Health, Publication guidelines | Assess simple descriptive and inferential statistics that fit the study design chosen and answer research question | **Compute** **descriptive and simple inferential statistics appropriate for the data and research question** |
| 15 | CTSA, Publication guidelines | Understand how to determine sample size, power, and precision for comparisons of two independent samples with respect to continuous and binary outcomes | **Understand the components of sample size, power, and precision** |
| 16 | Publication guidelines | **Understand statistical methods appropriate** to address loss to followup | **Understand the need** to address loss to followup |
| 17 | CTSA, Public Health, Publication guidelines | **Assess** the concepts and implications of reliability and validity of study measurements and evaluate the reliability and validity of measures | **Understand** the concepts and bias implications of reliability and validity of study measurements and evaluate the reliability and validity of measures |
| 18 | CTSA, Public Health | Assess the assumptions behind different statistical methods and their corresponding limitations and describe preferred methodologic alternatives to commonly used statistical methods when assumptions are not met | **Evaluate potential violations of the assumptions behind common statistical methods** |
| 19 | Publication guidelines | Identify inferential methods appropriate for clustered, matched, paired, or longitudinal studies | **Identify when clustered, matched, paired, or longitudinal statistical methods must be used** |
| 20 | First survey | Characterization of diagnostic testing, including sensitivity, specificity, and ROC curves | **Understand the concepts of sensitivity, specificity, positive and negative predictive value, and receiver operating characteristic curves** |
| 21 | CTSA | **Defend the significance** of data and safety monitoring plans. | **Understand the purpose** of data and safety monitoring plans |
| 22 | Publication guidelines | Identify adjusted inferential methods appropriate for the study design, including examination of interaction | **Identify appropriate methods to address potential confounding and effect modification** |
| 23 | CTSA | Understand the uses of meta-analytic methods | **Understand the purpose of meta-analysis and its place in the hierarchy of evidence** |
| 24 | CTSA, Publication guidelines | **Explain** the uses, importance, and limitations of early stopping rules in clinical trials | **Understand** the uses, importance, and limitations of early stopping rules in clinical trials |

*Sources of competencies:

Public Health: Calhoun et al (2008)

CTSA: CTSA Education Core Competency Workgroup.

Publication Guidelines: Enders (2011)

First survey: Oster et al. (2015)

BERD SIG: The Association for Clinical and Translational Science’s Biostatistics, Epidemiology, and Research Design Special Interest Group, teleconference held February 12, 2015 (unpublished)
