# Supplementary figures and images for: Statistical competencies for medical research learners: What is fundamental?
Source: J Clin Transl Sci. 2017 May 9;1(3):146–52. doi: 10.1017/cts.2016.31 (PMC5647667; doi:10.1017/cts.2016.31)

Appendix 1: Bar charts for all competencies, ordered as in Table 2.


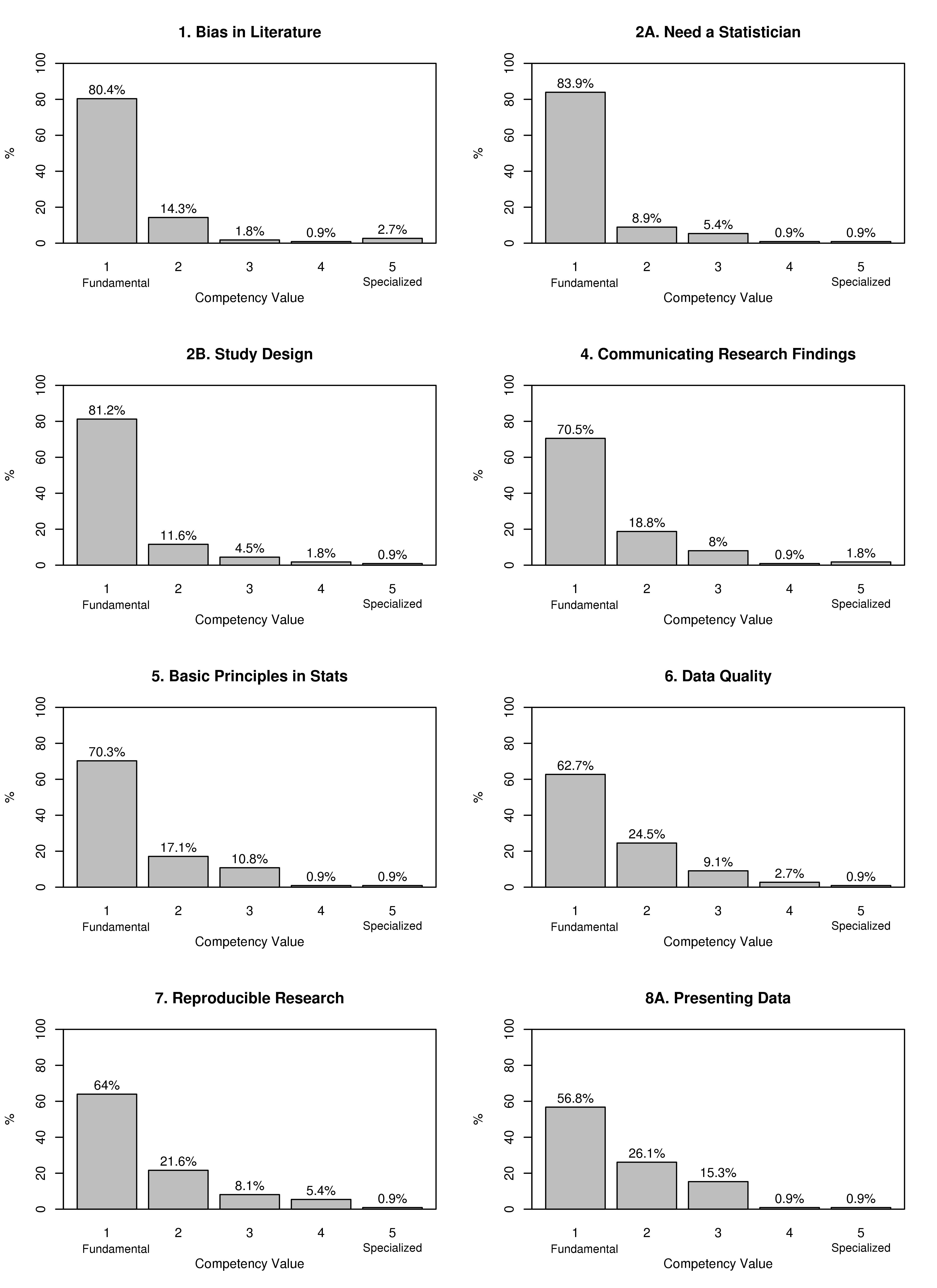

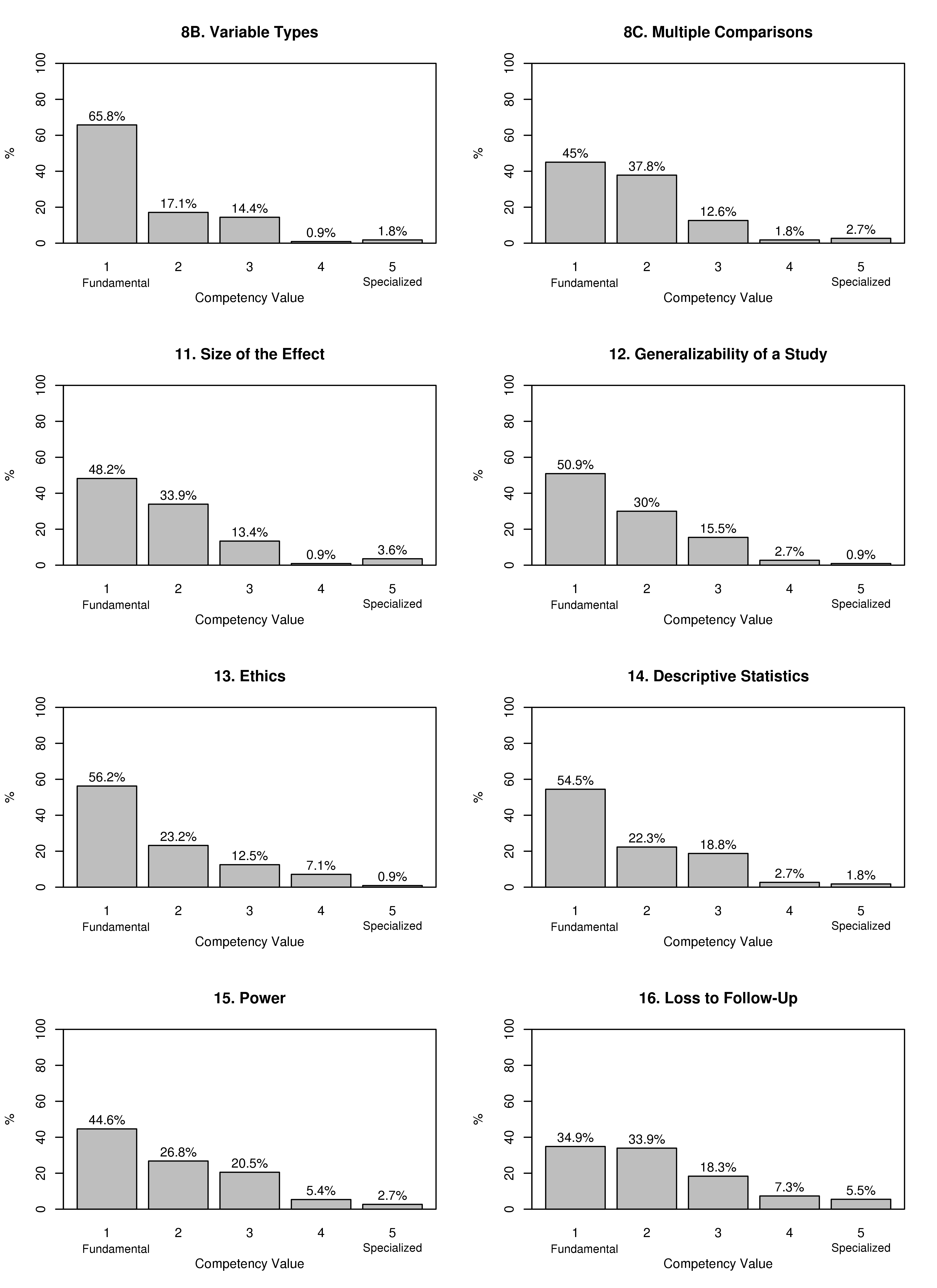

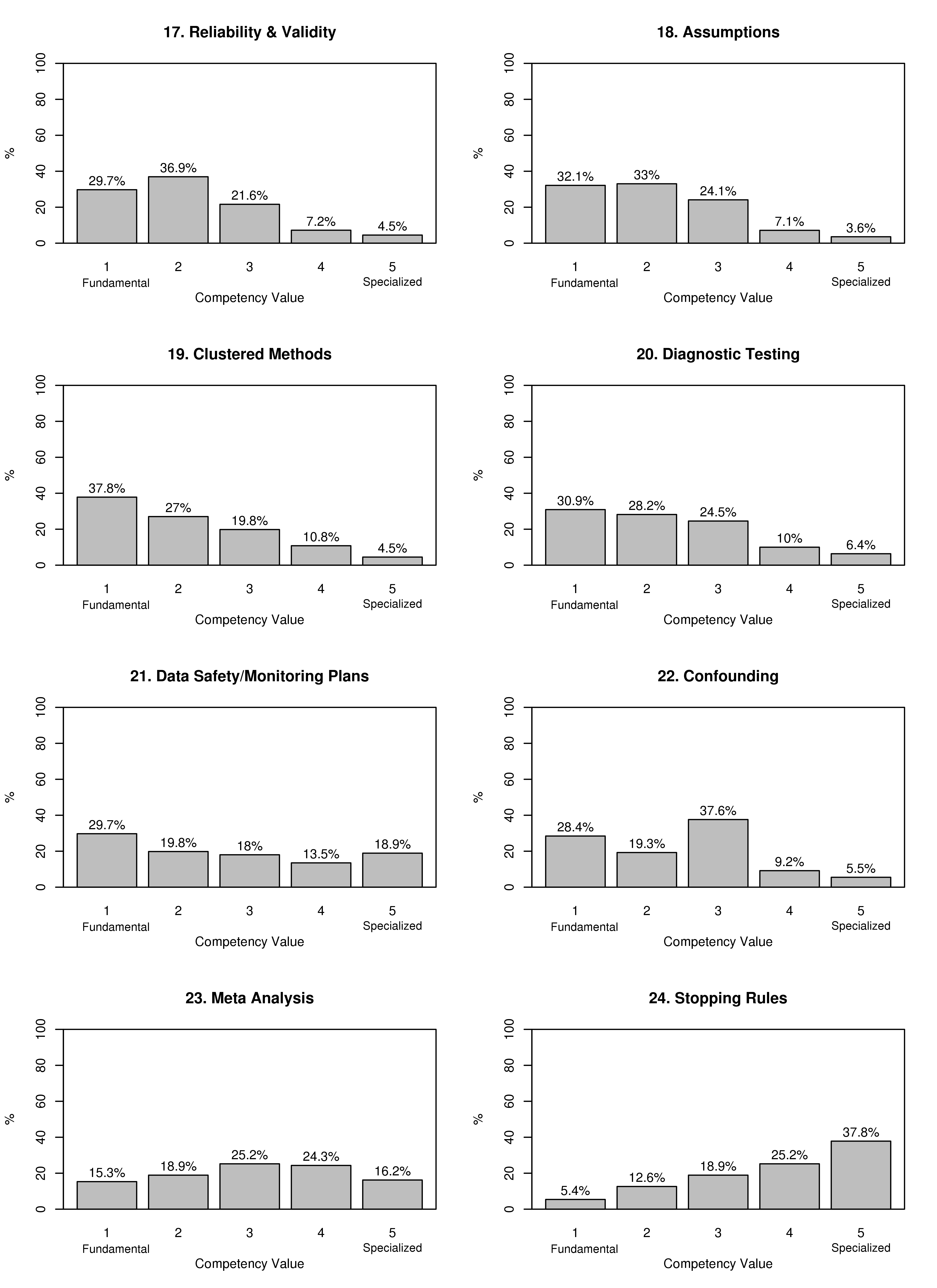

Supplement: Supplementary file 1 [file S2059866116000315sup.zip › S2059866116000315sup001.docx]
